# Eculizumab in the Treatment of Acetylcysteine-Induced Atypical Hemolytic Uremic Syndrome

**DOI:** 10.7759/cureus.37784

**Published:** 2023-04-18

**Authors:** Arielle Jalbert, Han Yao, Mylène Fagnan, Benoît Crevier

**Affiliations:** 1 Internal Medicine Core Program, Université de Sherbrooke (Faculty of Medicine and Health Sciences), Sherbrooke, CAN; 2 Nephrology, Centre Intégré de Santé et de Services Sociaux de la Montérégie-Centre, Greenfield Park, CAN; 3 Critical Care Medicine, Centre Intégré de Santé et de Services Sociaux de la Montérégie-Centre, Greenfield Park, CAN; 4 Pharmacy, Centre Intégré de Santé et de Services Sociaux de la Montérégie-Centre, Greenfield Park, CAN

**Keywords:** eculizumab, hemolysis, atypical hemolytic uremic syndrome, medication error overdose, acetylcysteine

## Abstract

N-acetylcysteine overdose is almost exclusively an iatrogenic event. This rare complication can lead to hemolysis or atypical hemolytic uremic syndrome. A 53-year-old Caucasian male accidentally received a two-fold N-acetylcysteine overdose that resulted in a presentation compatible with the atypical hemolytic uremic syndrome. The patient required temporary hemodialysis sessions, and he received treatment with eculizumab. This case report is the first reported N-acetylcysteine-induced atypical hemolytic uremic syndrome successfully treated with eculizumab. Clinicians should be aware of N-acetylcysteine overdose and its possible hemolytic complications.

## Introduction

Acetaminophen poisoning is the most frequent cause of liver transplantation in the United States of America. It was reported as responsible for 56,000 emergency department visits, 2600 hospitalizations, and 500 deaths per year in the U.S. [1]. The treatment of acetaminophen overdose is intravenous (IV) N-acetylcysteine (NAC) administered for at least 21 hours, and dosing is according to weight [1]. Due to the complexity of a multi-step administration regimen, IV NAC can be associated with medication errors [2-7].

We report the case of a patient who developed atypical hemolytic uremic syndrome (aHUS) after an NAC overdose due to a medication error. To our knowledge, this is the first probable NAC-induced aHUS successfully treated using the C5 complement inhibitor monoclonal antibody eculizumab.

## Case presentation

This case report presents a 53-year-old Caucasian male with no past medical history other than mild, untreated psoriasis and an episode of renal colic caused by urinary lithiasis more than a decade ago. He did not take any medication regularly at home, and he did not smoke, drink alcohol, or take recreational substances. He had no known medication allergies. The patient weighed 79.2 kg, and he was 168 cm tall. Bloodwork for a routine family doctor examination was normal approximately two years before the event. The patient consulted the emergency department with symptoms of persistent fever, dark urine, and an icteric complexion for seven days. He complained of fatigue, anorexia, and a decrease in urine output. He also noticed a slight dry cough and a frontal headache. The patient denied having diarrhea, abdominal pain, or other acute gastrointestinal symptoms. The history was remarkable for an intake of acetaminophen 1 g four times a day in addition to regular acetaminophen suppository use, cumulating to an approximate acetaminophen daily dose of 6 g per day (75 mg/kg/day) for the past seven days, which the patient took to decrease a fever episode. On physical examination, the patient was eupneic but diaphoretic. Vitals were as follows: blood pressure 177/108 mmHg, oxygen saturation of 96% on room air, pulse rate of 101 beats per minute, and a rectal temperature of 38°C. The patient had visible cutaneous and conjunctival icterus. On auscultation, the patient’s heart sounds were normal, accompanied by a 1/6 systolic murmur. The lung and abdominal examinations were unremarkable. He had no edema. Initial laboratory results, drawn at presentation (Day 0), and according to the patient’s clinical evolution, are displayed in Table 1. Of note, serum aminotransferase concentrations were elevated, with alanine transaminase (ALT) levels at 617 U/L and total bilirubin levels of 36 mcmol/L. Serum acetaminophen levels were less than 20 mcg/mL. All viral hepatitis serologies (hepatitis A, B, and C) were negative. Although the lactate dehydrogenase (LDH) and bilirubin were elevated, hemolysis was not suspected because he had a complete blood count with values in the normal range (Table 1). Given the clinical presentation, the diagnosis of an acetaminophen intoxication leading to the liver failure was made. Before the results of serum acetaminophen levels were available, IV NAC was administered according to a one-bag regimen of 150 mg/kg bolus in one hour then 15 mg/kg/hour for 20 hours at Day 0, i.e., the total NAC prescribed was 35.7 g in 21 hours. However, due to a pump programming error, the patient erroneously received NAC 70.8 g (1830 mL of a 38.7 mg/mL solution) administered in six hours and 45 minutes. This represents an overdose of two times the standard therapeutic dose over 21 hours. Although there was an improvement in the aminotransferase concentrations, the patient developed sudden onset thrombocytopenia, with platelets decreasing from 183 to 34 x10^9 cells/L, in addition to acute kidney injury (creatinine rising from 88 to 292 mcmol/L). With the ongoing deterioration of the renal function, the patient was transferred from a primary care hospital to a university-affiliated center for acute hemodialysis and an evaluation for plasma therapy. Further investigations showed that the urinary sediment contained red blood cells with many granular casts. A chest X-ray revealed slight pulmonary edema. Hemodialysis was initiated in the first 24 hours of transfer (Day 2). aHUS was suspected given that the patient showed biochemical and hematological signs of thrombotic microangiopathy: the peripheral blood smear showed the presence of schistocytes and anisopoikilocytosis, elevated serum levels of LDH of 2979 U/L, and progressive thrombocytopenia. Serum C3 and C4 levels were normal (1.07 g/L and 0.23 g/L, respectively). ADAMTS-13 levels were sent and resulted in 68%, ruling out typical thrombocytopenic thrombotic purpura and reinforcing the hypothesis of an aHUS. Microbiologic analyses for *Salmonella, Shigella, Yersinia enterocolitica, Campylobacter, Escherichia coli 0157*, malaria, *Borrelia burdorferi*, syphilis, Leptospira, parvovirus B19, Cytomegalovirus (CMV), and HIV were negative. Blood, stool, and urine cultures were negative. Glucose-6-phosphate dehydrogenase (G6PD) testing was normal. Plasma therapy was not initiated due to the rapid preliminary results of ADAMTS-13, the absence of Shiga-toxin, and the suspected cause of NAC poisoning. IV eculizumab 900 mg was initiated weekly for four doses, followed by 1200 mg every two weeks starting from the fifth week. Meningococcal immunization and penicillin prophylaxis were administered.

**Table 1:** Laboratory results according to the patient’s clinical evolution INR: international normalized ratio; aPTT: activated partial thromboplastin time; eGFR: estimated glomerular filtration rate; ALT: alanine transaminase; AST: aspartate aminotransferase; ALP: alkaline phosphatase; LDH: lactate dehydrogenase

Laboratories	Day 0 (at the primary care hospital)	Day 1	Day 2 (hemodialysis initiation)	Day 3 (day of eculizumab initiation)	Day 10 (7 days post-eculizumab initiation)	Day 91
Hemoglobin (g/L)	134	108	110	92	81	122
White blood cells (x 10^9 cells/L)	6.2	10.8	10.2	8.0	11.3	7.7
Platelet count(x10^9 cells/L)	183	34	51	52	541	409
INR		1.04	0.82			
aPTT (seconds)		55.9	26			
Fibrinogen (g/L)		2.52	3.75			
Serum creatinine (mcmol/L)	88	292	677	636		309
eGFR (mL/min/1.73m^2^)	86	20	7	8		19
Urea (mmol/L)			28.8	24.3	26.5	18
Albumin/creatinine (mg/mmol)						6.6
ALT (U/L)	617	397	171	138		
AST (U/L)		357	103	62		
Total bilirubin (mcmol/L)	36	39	32	30	12	
Conjugated bilirubin (mcmol/L)	21.1	22.7	22			
ALP (U/L)	232	148	99	100		
LDH (U/L)	516		2979	2300	710	

The patient’s liver function continued to improve six days post-NAC administration (Table 1). Initial aminotransferase elevation and hyperbilirubinemia were attributed to chronic acetaminophen intoxication. A week following the first dose of eculizumab, the platelet count gradually improved to 541x10^9 cells/L, and haptoglobin and LDH levels normalized (0.98 g/L and 710 U/L, respectively). The patient was discharged from the hospital on Day 10. He remained on outpatient intermittent hemodialysis three times a week at his primary care hospital. Hemodialysis treatments were discontinued about three months after the first eculizumab dose. His baseline serum creatinine level without renal replacement therapy stabilized at 309 mcmol/L (eGFR CKD-EPI=19 mL/min/1.73m^2^) at three months after eculizumab and at 179 mcmol/L (eGFR CKD-EPI=36 mL/min/1.73m^2^) a year after the end of hemodialysis. Eculizumab was discontinued approximately five months after its initiation.

The patient provided informed consent for the publishing of this case report.

## Discussion

Through research, efforts are made to simplify the IV NAC posology to avoid medication errors [8]. Even though NAC is known to have an advantageous safety profile, rare case reports of serious adverse effects, including death, have previously been published [3]. NAC overdose was reported as a potential causative agent for aHUS and hemolysis [4-7]; it is a serious condition that can result in mortality [5]. The mechanism of NAC overdose-induced hemolysis is not known. Some of the proposed mechanisms of toxicity are related to the hyperosmolar nature of the IV NAC solution or a direct effect on erythrocyte metabolism (i.e., inhibited oxidation of nicotinamide adenine dinucleotide phosphate (NADPH) with high concentrations of cysteine and inhibition of glutathione reductase leading to disruption of the pentose phosphate pathway; a result may be oxidative stress for erythrocytes) [6]. Eculizumab is a first-line treatment for patients with aHUS [9]. The treatment described sheds new light on the possible treatment for this rare iatrogenic condition. Also, to our knowledge, this is the first case of a probable aHUS associated with a NAC overdose from a one-bag NAC regimen.

Considering the timing of the adverse event, the dose of NAC administered, the sudden clinical presentation, and previous reported case reports of hemolysis [4-7], the causality of the NAC overdose in this case report and aHUS is highly plausible. Also, the fact that the patient did not take any other drugs known to induce hemolysis strengthens the causality link between NAC overdose and aHUS. The absence of G6PD deficiency, as shown in this case report, is compatible with NAC overdose, aHUS, or hemolysis [5,6]. In this case report, eculizumab associated with hemodialysis resulted in a rapid clinical and biochemical response, as demonstrated in Table 1.

There are some limitations to this case report. The Naranjo Algorithm (Adverse Drug Reaction Probability Scale) was used to determine a causal relationship between the NAC overdose and aHUS. The total score was five; the interpretation of the score indicates a probable causality relationship. Nevertheless, the Naranjo Scale in overdose patients is not validated; it was designed to assess the probability an event was caused by the administration of a drug at therapeutic posology [10]. Another limitation is that initial acetaminophen intoxication was not confirmed with acetaminophen-protein adducts, thus acetaminophen intoxication could be qualified as uncertain. In retrospect, it would have been useful to see if there were schistocytes on the blood smear before NAC administration to prove there was no hemolysis before or at Day 0.

## Conclusions

This case report presents a 53-year-old Caucasian male treated with eculizumab for aHUS following a two-fold NAC overdose due to a medication error. Hemolysis or aHUS attributed to NAC overdose is a rare iatrogenic condition described in some case reports. The treatment for this patient’s condition was abrupt discontinuation of NAC, hemodialysis for severe renal failure, and eculizumab. To our knowledge, this is the first case report of aHUS attributed to a NAC overdose treated with eculizumab with favorable outcomes such as rapid reversal of hemolysis, reversal of thrombocytopenia, and renal recovery (i.e., end of hemodialysis after three months). Clinicians should be aware of NAC overdose and the possible consequence of hemolysis.
